# Co-targeting of HMG-Co-A Reductase and Cycloxygenase-2 for the Treatment of Non-small Cell Lung Cancer

**DOI:** 10.34172/apb.2024.026

**Published:** 2024-01-13

**Authors:** Ashutosh Sharma, Ammu V.V.V. Ravi Kiran, Praveen T. Krishnamurthy

**Affiliations:** Department of Pharmacology, JSS College of Pharmacy (JSS Academy of Higher Education & Research), Ooty, The Nilgiris, Tamil Nadu 643001, India.

## To Editor,

 Non-small cell lung cancer (NSCLC) accounts for about 80-85% of total lung cancer cases with survival rate of <5 years, worldwide.^1^ The current therapeutic strategies for NSCLC include surgery, radiation therapy, chemotherapy and immunotherapy, limited due to their off-target side effects and patient incompliance.^2^

 Among many cellular metabolic pathways, Mevalonate pathway is crucial metabolic process, due to its involvement in cellular energetics. HMG-Co-A reductase is a key enzyme which converts 3-hydroxy-3methylglutryl -CoA (HMG-Co-A) to mevalonate and further to farnesyl pyro-phosphate (FPP) geranylgeranyl pyro-phosphate (GGPP). HMG-CO-A expression has been associated with various cancers, such as breast, ovarian and colorectal cancer. Several studies have shown that high HMG-CO-A expression is associated with increased tumor progression and metastasis in clinical scenario. Moreover, inhibition of HMG-CO-A activity reduces the production of cholesterol and other isoprenoids which could affect their metabolic’s and starve the tumors. Statins have been reported to exert anti-tumoral effects by modulating cell proliferation, apoptosis, angiogenesis and metastasis. Therefore, HMG-CO-A expression may serve as a potential therapeutic target in cancer. Inhibition of Mevalonate pathway by HMG-Co-A reductase inhibitors could help in blocking the cell cycle check points, resulting in blocking of NSCLC proliferation.^3^

 Moreover, upregulation of HMGCO-A in tumors improves inflammatory mediators such as cyclooxygenase-2 (COX-2) which is responsible for the angiogenesis, metastasis via thromboxane mediated PI3K/AKT activation.^4,5^ Blocking of COX-2 enzyme via COX-2 inhibitors such as celecoxib could be synergistic/additive effect in combination with HMG-Co-A reductase inhibitors.

 Co-delivery of conventional chemotherapeutics such as paclitaxel, doxorubicin, etc. along with non-oncological agents i.e., HMG-Co-A reductase inhibitors along with COX-2 inhibitors in NSCLC could not only improve the synergetic anticancer effects but also improve therapeutic outcomes. Further, repurposing of existing FDA approved drugs can expedite the drug development process, reduce costs, and potentially uncover new treatment options for various types of cancer. This innovative approach leverages the extensive knowledge and safety profiles of existing drugs, making it a valuable avenue for discovering novel cancer treatments and improving patient outcomes.^6^

## Competing Interests

 The authors state that there is no competing/conflict of interest.

## Ethical Approval

 Not applicable.
